# Comparative analysis of soil organic matter fractions, lability, stability ratios, and carbon management index in various land use types within bharatpur catchment, Chitwan District, Nepal

**DOI:** 10.1186/s13021-023-00241-1

**Published:** 2023-11-03

**Authors:** Yves Theoneste Murindangabo, Marek Kopecký, Trong Nghia Hoang, Jaroslav Bernas, Tulsi Parajuli, Suman Dhakal, Petr Konvalina, Jean de Dieu Marcel UFITIKIREZI, Gisele Kaneza, Babu Ram Khanal, Shiva Chandra Dhakal, Arjun Kumar Shrestha

**Affiliations:** 1 Faculty of Agriculture and Technology, University of South Bohemia in České Budějovice, 37005 České Budějovice, Czech Republic; 2 Faculty of Agriculture, Agriculture and Forestry University, Rampur, Chitwan Nepal; 3 University of Agriculture and Forestry, Hue University, 102 Phung Hung, Hue City, Vietnam

**Keywords:** Soil carbon pools, Soil carbon management, Soil degradation indicators, Land use/Land cover change, Soil organic matter fractions

## Abstract

**Background:**

Land use and land cover changes have a significant impact on the dynamics of soil organic matter (SOM) and its fractions, as well as on overall soil health. This study conducted in Bharatpur Catchment, Chitwan District, Nepal, aimed to assess and quantify variations in total soil organic matter (T_SOMC_), labile organic matter fraction (C_L_), stable organic matter fraction (C_S_), stability ratio (SR), and carbon management index (CMI) across seven land use types: pastureland, forestland, fruit orchards, small-scale conventional agricultural land, large-scale conventional agricultural land, large-scale alternative fallow and conventional agricultural land, and organic farming agricultural land. The study also explored the potential use of the Carbon Management Index (CMI) and stability ratio (SR) as indicators of soil degradation or improvement in response to land use changes.

**Results:**

The findings revealed significant differences in mean values of T_SOMC_, C_L_, and C_S_ among the different land use types. Forestland and organic farming exhibited significantly higher T_SOMC_ (3.24%, 3.12%) compared to fruit orchard lands (2.62%), small scale conventional farming (2.22%), alternative fallow and conventional farming (2.06%), large scale conventional farming (1.84%) and pastureland (1.20%). Organic farming and Forestland also had significantly higher C_L_ (1.85%, 1.84%) and C_S_ (1.27%, 1.39%) compared to all other land use types. Forest and organic farming lands showed higher CMI values, while pastures and forests exhibited higher SR values compared to the rest of the land use types.

**Conclusions:**

This study highlights the influence of various land use types on soil organic matter pools and demonstrates the potential of CMI and SR as indicators for assessing soil degradation or improvement in response to land use and land cover changes.

## Background

Anthropogenic disturbances, particularly land use/cover change, have been recognized as major contributors to soil quality deterioration worldwide [1]. SOM has gained increasing attention in soil quality assessment due to its multifaceted impact on soil chemical, physical, and biological properties [2]. Despite being considered one of the least understood components of soil due to its dynamic nature [3], SOM has been linked to its potential role in soil quality improvement and carbon sequestration through effective management of land use and cover types [4]. Land use and cover types influence carbon fluxes in ecosystems through factors such as litter quality, deposition, and turnover rates. While SOM serves as an indicator of soil quality, the conceptualization of SOM fractions provides a valuable approach to detect even the short or long duration subtle changes in management practices and regulate degradation [5, 6].

Various techniques are employed to partition SOM fractions into its functional pools. In this study, we employed chemical fractionation based on organic matter carbon oxidizability. The use of SOM degrees of oxidation allowed us to separate SOM labile and stable fractions, as demonstrated in other studies [5, 7, 8]. This methodology involved evaluating the reaction kinetics during the K_2_Cr_2_O_7_ oxidation of SOM in H_2_SO_4_ at different temperatures and durations. The total carbon content was determined by catalytic oxidation of the sample at 1100 °C. Soil organic matter can be categorized into different fractions based on their levels of stability or lability. The labile fraction (C_L_) is particularly noteworthy due to its high turnover rate and vulnerability to management systems and erosion [8]. It plays a crucial role in determining the nutrient regime of a specific soil and provides essential nutrients and substrates for soil microorganisms [9]. Soil scientists have described the labile fraction in various ways, including particulate organic carbon (POC) (53–2000 µm), light fraction organic carbon (LFOC) (density of < 2.0 g cm^−2^), readily oxidized carbon (ROC), soil microbial biomass carbon (SMBC), and dissolved organic carbon (DOC) [10, 11]. The labile fraction primarily consists of mineral-free SOM composed of partially decomposed plant and animal residues that rapidly turn over and have a lower specific density compared to soil minerals [12]. Agricultural soils typically exhibit lower C_L_ due to intensive disturbances from tillage practices and crop residue removal [13]. In contrast, native land cover types such as forests, grasslands, and shrublands exhibit higher C_L_ due to increased litter inputs, reduced soil disturbances, and controlled soil temperature and moisture [6]. In pastures, grazing activities have been observed to enhance carbon lability through microbial activation by enzymes present in herbivore saliva and dung, particularly in warm temperatures [14, 15]. Furthermore, biomass removal promotes plant regrowth, thereby facilitating nutrient cycling within the rhizosphere. With increasing grazing intensity, C_L_ tends to decrease significantly due to reduced litter deposition, heightened mineralization from surface temperature exposure, and intensive erosion [16].

On the other hand, the stable fraction (C_S_) represents the higher portion of the total organic matter [17] and exhibits resistance to oxidation and decomposition, playing a pivotal role in the long-term storage of carbon within the soil. While some studies suggest that C_S_, owing to its recalcitrant nature, is minimally affected by land use or management practices [18], others indicate that this fraction may be more susceptible to change compared to the labile portion [19, 20]. The C_S_ is often regarded as resistant to various management systems. This resilience stems from its inherent protection against external factors, achieved through sorption onto fine soil particles. This sorption process involves the secure attachment of the stable fraction of soil organic matter to these fine particles. By doing so, it shields this stable fraction from the influence of external factors and microbial decomposition. This adsorption process serves as a safeguard, ensuring that the stable organic matter remains bound to soil particles, thus impeding its immediate decomposition. Consequently, this contributes significantly to the formation of stable fraction and humus, the dark and organic component of soil. Furthermore, the recalcitrant nature (the component of SOM that is resistant to microbial decomposition or protected by mineral soil particles) of C_S_ further reinforces its inaccessibility to decomposing microbes, thereby playing a pivotal role in the intricate stabilization and humification processes [21]. Therefore, finding out the lability of SOM within each land use/ cover type can serve as an early indicator of soil degradation or improvement in response to different management practices.

To enhance the sensitivity of soil quality assessments across diverse land uses, the incorporation of more refined indicators such as the carbon management index (CMI), and stability ratio (SR) has been employed. These indicators offer valuable insights into the management, lability, and stability of soil carbon across various land use practices [5, 22]. The CMI provides insights into the capacity of a land use type to promote soil quality and the SR represents the percentage of the stabilized fraction in relation to the total organic matter carbon content in the sample [23, 24].

Studies utilizing the CMI and SR as assessment tools are relatively scarce, particularly none has been conducted at this study location. Therefore, the objective of this study is to investigate the dynamics of SOM pools in different land use and land cover types within the Bharatpur Catchment, Chitwan District, Nepal, and develop a CMI to assess the soil quality there. By assessing the CMI and SR, we can gain a better understanding of the stability and turnover dynamics of SOM in Bharatpur Catchment area, which are crucial for soil health and carbon cycling processes. These parameters provide valuable information for evaluating soil quality and guiding sustainable land management practices.

## Methods

### Description of study site

The study was conducted in the Bharatpur catchment, located in the Chitwan District, which is situated in the southwestern corner of Bagmati Province in southern-central Nepal. The district is positioned between 27° 35′ 0″ N and 84° 30′ 0″ E. The topography of the area is terai, with an elevation of approximately 208 m above sea level. The catchment is situated within the plain ecological zone and experiences a humid subtropical monsoon climate, classified as Cwa according to Köppen and Geiger [25].The region has high humidity throughout the year, characterized by mild and generally warm temperatures. The average high temperature in Bharatpur ranges from 24 °C to 30 °C, with occasional peaks reaching up to 35 °C, and a record high temperature of 46 °C. On the other hand, the minimum temperature hovers around 14.05 °C, with a record low of 2 °C. On an annual basis, the average temperature in Bharatpur stands around 29.28 °C, which is 7.28% higher than the overall averages of Nepal [26, 27]. The annual rainfall is approximately 2407 mm, with the majority occurring during the summer months of June and July. The remaining months receive scattered rainfall, particularly during the winter (November–December) and pre-monsoon (February–March) periods. The Bharatpur catchment is located on the banks of the Narayani River and is bordered by the young chure hills, which are prone to erosion [28, 29].

Renowned scholar William G. Axinn [30] highlights the Chitwan Valley, including the Bharatpur catchment, as a truly remarkable place in Nepal. Despite its relatively low altitude, the valley remained secluded and cut off from the outside world until the mid-1950s, ensconced by impenetrable jungles and formidable predators. However, in the late 1950s, Nepal’s government embarked on an ambitious endeavor to clear vast expanses of the jungle and allocate the land to settlers from the hills and mountains. By the mid-1970s, aided by the support of various international donor organizations, the Nepalese government implemented extensive programs aimed at fostering social and economic transformation in Chitwan. The impact was profound, as witnessed by the residents of Chitwan during the 1990s, who experienced a remarkable metamorphosis in their communities. Once isolated, rural, and reliant on subsistence agriculture, these neighborhoods evolved into vibrant, educated, and market-oriented hubs seamlessly connected to the broader global systems. Undoubtedly, this shift brought about significant alterations in terms of land use and land cover, signifying a compelling and dynamic narrative of change.

Furthermore, our study area enjoys relative climate uniformity, with close proximity and similar levels of temperature and precipitation across all assessed lands. This climate homogeneity diminishes the likelihood of climate being the primary catalyst for variations in soil organic matter among land use types. Despite comparable climate conditions, the management, retention, and practices related to these climatic elements vary significantly among the different land use types, emphasizing the role of human activity. Lastly, considering the historical context, substantial land use changes have transpired since the 1950s when government-led land distribution transformed forested areas into the diverse land use patterns we observe today. This historical transformation underscores the pivotal role of land use practices in driving soil organic matter changes, even in the context of consistent climate conditions.

### Land use

Chitwan District exhibits diverse land use types influenced by factors such as population, socioeconomic development, climate, topography, and forest management [30]. An analysis of the land use/land cover using Landsat imagery from 2020 revealed that the Chitwan area consists of grassland (1.73%), barren areas (1.76%), riverine forests (1.93%), water bodies (1.97%), developed areas (4.13%), Sal-dominated forests (15.4%), croplands (28.13%), and mixed forests (44.95%). Comparing land cover changes between 2000 and 2020, there was an overall increase in Sal-dominated forests (7.6%), developed areas (31.34%), and mixed forests (37.46%), and a decrease in riverine forests (11.29%), barren areas (20.03%), croplands (29.87%), and grasslands (49.71%) [31, 32]. A significant portion of the forested land in the district is the Chitwan National Park, which is protected as a habitat for various wildlife and other ecological benefits, and attracts a large number of tourists [33]. The grasslands and pastures primarily cater to domestic buffalo, dairy cattle, sheep, goats, and other wild animals such as rhinoceros. The proportion occupied by crop and grass lands (agro-pastoralism) depends on population growth and urbanization in the district (3^rd^ after Kathmandu and Pokhara) in recent years, which has also impacted other land use types [30]. Farming practices in the area involve intercropping or crop rotation, with rice and wheat as the main crops, along with legumes such as soybean, common bean, black gram, green gram, and cluster bean, oilseeds like rapeseed and sunflower, and various vegetables and tropical fruits including carrots, tomatoes, green peppers, onions, lychee, mango, pineapples, banana, papaya, guava, and citrus [34].

In our study, we primarily examined land use types and organic matter dynamics in the designated region. Based on the 2019 pilot agriculture integrated survey for Chitwan, the total agricultural area is 33,836.23 hectares (orchards: 1784.21 ha, pastures/meadow: 155.44 ha, alternative fallow: 446, 79 ha, organic farming and other agricultural activities: 846.74 ha, small-scale and large scale farming: 30603.5 ha while other areas cover 5172 hectares, including forests (346.61 ha)) and ponds [35]. Including these categories was crucial for a comprehensive assessment, providing insights into their ecological and environmental impacts.

### Bharatpur soils

The Bharatpur plain area is characterized by fertile alluvial soils with high agricultural potential. The soil has a sandy-loam texture, is well-drained, and relatively deep. The soil composition shows slight variations, with approximately 57–66% sand, 15–24% silt, and 11–18% clay content. The soils in the area are generally acidic, regardless of the land-use system, with a soil pH of around 5.4 [26, 36].

### Sampling design and soil sampling

The samples were meticulously collected concurrently in March 2023. The sampling method involved dividing the study area into representative subplots for each land use type. A composite soil sample consisted of 15 to 20 sample sites per field, with at least one sampling site for every 2 to 4 hectares, depending on the typical characteristics and size of the land use type. This approach aimed to capture the variability within the land use type while avoiding impracticality in collecting samples from multiple locations within it. Within each plot, soil samples were collected using an auger from the center as well as near the four corners, at a depth of 20 cm. This sampling strategy captured representative soil characteristics across the entire plot. The individual core samples obtained from each land use type were thoroughly mixed together to create a composite sample. Approximately 600 g of soil was bulked from each composite sample. These composite samples were then dried, ensuring the removal of moisture from the soil and providing a consistent basis for subsequent analyses [37]. During the sampling process, the geographical position of each plot was recorded to establish spatial reference points for the study area and the average altitude of all land use where samples were taken is 208 m absl. The selected land use types and practices have been established for a minimum of 30 years (since 1990), ensuring a long-term representation of the different land management approaches. The specific details of each land use type are as follows:

A. Large scale conventional farming soil sample of sandy-loam at Bharatpur locality, 228 m above sea level, GPS coordinates: 27°38′49.4"N, 84°20′57.0"E. The soil belongs to the large-scale conventional farming area within the Agriculture and Forestry University, Rampur, Chitwan farm. This area, covering approximately 200 hectares, is divided into agricultural fields and fruit orchards dominated by lychee (litchi) and mango. Conventional farming practices are employed in this part of the farm, including the cultivation of mainly wheat, rice, and few vegetables using synthetic fertilizers and chemicals.

B. Alternative fallow and conventional (large scale) farming soil sample of sandy-loam at Bharatpur locality, 228 m above sea level, GPS coordinates: 27°38′45.2"N, 84°20′53.5"E. The soil is a section within the Agriculture and Forestry University, Rampur, Chitwan farm where alternative fallow practices are implemented along with conventional farming. This area is used for the rotation of crops such as legumes (soybean, common bean, cluster bean, mungbean, blackgram) and oilseeds (Rapeseed, Sesame, sunflower) alternating with fallow periods. Conventional farming practices, including soil disturbances and the use of synthetic fertilizers and chemicals, are employed.

C. Fruits orchard soil sample of sandy-loam at Bharatpur locality, 228 m above sea level, GPS coordinates: 27°39′07.3"N, 84°21′06.0"E. The soil represents the fruit orchard area within the Agriculture and Forestry University, Rampur, Chitwan farm. The orchard is primarily composed of lychee (litchi) and mango fruits with sometimes pineapple as the filler crop. Orchard management include minimum soil disturbance during weeding and occasional fertilizers application.

D. Small scale conventional farming soil sample of sandy-loam at Bharatpur locality, 200 m above sea level, GPS coordinates: 27°38′24.4"N, 84°22′18.8"E. The soil belongs to small-scale farmers located near the Agriculture and Forestry University, Rampur, Chitwan farm. The small scale farmers cultivate mainly crops such as wheat, rice, maize, and various vegetables using conventional farming practices that involve soil disturbances and moderate use of inputs (organic or/and synthetic). Rice–Wheat-Vegetables and Rice-Rapeseed-Vegetables are the dominant cropping systems adopted here.

E. Pastureland (Gyaneshwor) soil sample of sandy-loam at Bharatpur locality, 180 m above sea level, GPS coordinates: 27°41′31'' N, 84°20′9'' E. It represents pastures used for livestock grazing by domesticated animals such as buffalo, cattle, goats, and sheep of nearby farmers. These pastures are also occasionally accessed by some wild animals, including Rhinoceros. F. Forestland (Gyaneshwor Community Forest) soil sample of sandy-loam at Bharatpur locality, 180 m above sea level, GPS coordinates: 27°41′30'' N, 84°20′26'' E. The soil belongs to a community forest located adjacent to the pastures. This forested area is managed for conservation purposes, providing habitat for wildlife and promoting ecosystem stability. The soil in this area remains completely untouched by any form of cultivation or disturbances, closely resembling pristine natural soil conditions.

G. Organic farming soil sample of sandy-loam at Bharatpur locality, 200 m above sea level, GPS coordinates: 27°38′25'' N, 84°22′16'' E. The soil belongs to Organic Ghar: Agri Training Center, located near Agriculture and Forestry University, and is dedicated to organic farming practices. The center employs methods, which prioritize the use of natural inputs and techniques to promote soil health and sustainability. Natural methods of soil replenishment like crop rotation, green manuring, organic pesticides (some made at the center) are used along with local farmyard manure and compost (compost tea).

These specific locations were selected to represent different land use types within the study area, allowing for a comprehensive assessment of soil characteristics and dynamics across different management approaches.

We implemented a rigorous sampling protocol that maintained a minimum separation distance of at least 1.5 km between sampling sites. This approach was intentionally designed to minimize the potential influence of adjacent land use types on our samples and to ensure the robustness of our results.

### Chemical analysis

#### Total soil organic matter carbon (T_SOMC_)

The total organic matter carbon (T_SOMC_) content of the soil samples was determined using the Primacs SLC Analyzer (SKALAR, Netherlands). This analyzer features a dual-oven design that enables the separate analysis of total carbon (TC) and inorganic carbon (IC). The TC analysis involves catalytic oxidation of the sample at a high temperature of 1100 °C, converting the carbon in the sample into CO_2_. The CO_2_ produced is then detected by a nondispersive infrared detector. On the other hand, the IC analysis involves acidification of the sample in the IC reactor, which converts the inorganic carbon present into CO_2_. By subtracting the IC value from the TC value, the T_SOMC_ content is obtained $(\mathrm{TC }-\mathrm{ IC }= {\mathrm{T}}_{\mathrm{SOMC}})$. Additionally, other analyses were performed following specific protocols outlined below. Each analysis was repeated three times for each individual sample, ensuring reliable and accurate results [7, 8, 18].

#### Soil Organic matter fractions [stable fraction (CS), labile fraction (CL)]

The evaluation of the labile fraction (C_L_) and stable fractions (C_S_) of SOM was conducted separately to assess both their quantity and quality. The quality of the C_L_ was determined based on its oxidation speed constant (k), which provides insights into its decomposition rate [8, 22]. On the other hand, the quality of the C_S_ was expressed in terms of stable fraction Stability Ratio (SR), which offer information about its proportion and contribution to the long-term total organic matter. The method used for this evaluation is based on the principles described by Blair et al. and Ciavatta et al. [5, 23].

#### Quality and quantity of SOM labile fraction (CL)

To determine the quality and quantity of the C_L_ a procedure was followed. Soil samples were collected and processed as described earlier, and five flasks were prepared for each sample. The samples were then subjected to oxidation in a solution of 0.07 mol/L of K_2_Cr_2_O_7_ in 12 mol/L of H_2_SO_4_ at a temperature of 60 °C. Partial samples were taken out at regular intervals of 10 min, 20 min, 30 min, and 40 min during the oxidation process. The amount of oxidizable carbon (C_OX_) in the samples was determined by titration (automatic titrator DL 50 Mettler-Toledo, Greifensee, Switzerland). Then, the temperature was raised to 90° C and, after 30 min, C_OX_ was determined in the sample from the last flask and was designated as C_L._

Additionally, the constant k, which represents the speed of oxidation, was calculated for the C_L_. This involved comparing the differences between C_OX_ values obtained at different time intervals and C_L_, which was determined at the end of the oxidation process at 90 °C (30 min). The logarithms of these differences were plotted on a graph, with time (in minutes) on the x-axis and the logarithms on the y-axis. The slope of the trend line on the graph represented the ratio between the opposite and adjacent legs of a right-angled triangle (tg α), and the constant k was calculated as 2.303 times that ratio.

The calculated constant k, expressed in seconds for clarity, provides valuable information about the oxidation kinetics and quality of the labile fraction (C_L_) of SOM. A higher value of k indicates greater lability of C_L_, which signifies better quality in terms of its main functions in agricultural soil, such as serving as an energy source for soil organisms and a nutrient source for crops. However, it also signifies a high decomposition rate and carbon emission.

#### Quality and quantity of SOM stable fraction (CS)

When SOM is subjected to oxidation in a solution of 0.07 mol/L of K_2_Cr_2_O_7_ in 12 mol/L of H_2_SO_4_, only the C_L_ participates in the reaction, while the stable organic matter fractions remain unaffected. By determining the total soil organic matter carbon (T_SOMC_) in a soil sample, the difference between T_SOMC_ and C_L_ provides an estimation of the amount of the stable fraction of organic matter, referred to as C_S_.

The quality of the stable fraction of SOM was assessed using the stability ratio (SR) parameter. The SR represents the percentage of the stabilized fraction in relation to the total organic matter carbon content in the sample. It provides a measure of the degree of stabilization of organic matter in the soil, indicating the proportion of carbon that is resistant to decomposition. A higher SR value suggests a greater degree of stabilization and indicates that a larger proportion of organic carbon is less prone to rapid decomposition, thus potentially contributing to long-term carbon storage in the soil.

#### Carbon management and stability indices

The Carbon Management Index (CMI) is an evaluation model used to assess the impact of specific land uses on soil quality compared to a reference land use soil [22]. It takes into consideration the relationship between soil carbon supply and both the total pool size and lability (turnover rate). In order to derive an accurate carbon management index, it is crucial to consider both factors. The total pool size refers to the quantity of carbon present in the soil, while lability represents the rate at which carbon is being cycled and transformed within the soil system. Both aspects are important for understanding the dynamics of carbon supply and its impact on soil quality. They help identify land use practices that either enhance or hinder carbon management, thereby assisting in the development of sustainable soil management strategies [6, 13].

The Carbon Management Index (CMI) is calculated using the formula [5]:

$${\text{CMI}} = {\text{CPI}} \times {\text{LI}} \times \,100$$

where CPI represents the Carbon Pool Index and LI represents the Lability Index.

$$\mathrm{CPI}=\frac{\mathrm{Total \,Carbon\, in \,the \,sample }(\mathrm{treatment})}{\mathrm{Total\, Carbon\, in\, the\, reference}}$$

$$\mathrm{LI}=\frac{\mathrm{L \,of\, carbon\, in\, the\, soil\, sample }(\mathrm{treatment})}{\mathrm{L\, of\, carbon\, in\, the\, reference\, soil}}$$

where L is the soil carbon lability.

$$\mathrm{L \,of \,carbon}= \frac{\mathrm{Labile\, carbon\, in\, the\, soil \,sample }({\mathrm{C}}_{\mathrm{L}})}{\mathrm{Non}-\mathrm{Labile\, carbon\, in\, sample\, soil }({\mathrm{C}}_{\mathrm{S}})}$$

In this study, native forestland was chosen as the reference land use because it has remained undisturbed and preserved in its natural state since its establishment.

It should be noted that the loss of carbon (C) from a soil with a large carbon pool is less significant compared to the loss of the same amount of C from a soil that is already depleted of C or had a smaller initial C pool. Similarly, the more a soil is depleted of carbon, the more challenging it becomes to restore its carbon content.

The stability ratio (SR) was calculated using the following formulas [23]:

$$\mathrm{SR}=\frac{{\mathrm{C}}_{\mathrm{S}}}{{\mathrm{T}}_{\mathrm{SOMC}}} \times 100$$

With C_S_ representing the stable fraction of soil organic matter carbon, C_L_ representing the labile fraction of soil organic matter carbon, and T_SOMC_ representing the total soil organic matter carbon.

### Statistical analysis

The analysis of variance (ANOVA) and post hoc Tukey's honestly significant difference test for multiple comparisons of means were conducted using Statistica 14.0 software, TIBCO Inc., Palo Alto, CA, USA, 2021. Statistical significance was evaluated at a significance level of p < 0.05. A simple linear regression analysis was used to reveal the relationship between TOC and its fractions.

## Results

### Total soil organic carbon (%)

The total soil organic carbon (T_SOMC_) exhibited significant variation among all land use types (F (6, 14) = 883.44, P < 0.05), except for forestland, which showed no statistical difference compared to organic farming. Notably, forestland and organic farming land displayed the highest T_SOMC_ levels, measuring at 3.24% and 3.12% respectively. This disparity can be attributed to the minimum of disturbances and the continuous accumulation of organic litter in the forest land, as well as the longstanding utilization of organic inputs in the case of organic farming. On the other hand, pasture land and large-scale conventional farming land exhibited the lowest T_SOMC_ values, standing at 1.20% and 1.84% respectively. This can be attributed to factors such as overgrazing, increased exposure, and significant soil disturbances associated with conventional farming practices.

### Labile fraction of soil organic matter (%)

The labile fraction of soil organic matter (C_L_) demonstrated significant differences among all land use types (F (6, 14) = 7830.1, P < 0.05), except for organic farming, which showed no statistically significant difference when compared to forestland. Remarkably, organic farming and forest land showcased the highest C_L_ levels, measuring at 1.85% and 1.84%, respectively. Conversely, pasture land and large-scale conventional farming exhibited the lowest C_L_ values, measuring 0.67% and 1.11%, respectively.

### Stable fraction of soil organic matter (%)

The stable fraction of soil organic matter (Cs) exhibited lower mean values compared to the labile fraction across all land use types. Significant variations were observed in the Cs among all land use types (F (6, 14) = 4192.2, P < 0.05). Forestland displayed the highest Cs, measuring at 1.39%, while pasture land exhibited the lowest Cs, measuring 0.52%.

### Oxidation speed constant, *k* (*%*)

The speed of oxidation exhibited variations among all land use types (F (6, 14) = 81.377, P < 0.05). Notably, alternative fallow and conventional farming, along with large-scale conventional farming, did not show any statistically significant difference in terms of oxidation, similar to forest and pastureland. Conversely, fruit orchard land and organic farming displayed the lowest oxidation coefficients, measuring at 1.85% and 1.89%, respectively. On the other hand, large-scale conventional farming and alternative fallow and conventional farming land demonstrated the highest oxidation coefficients, measuring at 2.78% and 3.32%, respectively.

### Carbon management index and Stability Ratio

According to Table 1, the carbon management index demonstrates the highest values in organic farming and forest land, while the lowest value is observed in pasture land, with percentages of 96%, 100%, and 13% respectively. On the other hand, the stability ratio is higher in pasture and forest land, whereas it is the lowest in small-scale conventional farming, with percentages of 43%, 42%, and 30% respectively. For the purpose of this study, forest land was considered as the reference land use type.

**Table 1:** Effects of land use types on carbon management index and stability ratio

Land use type	L	LI	CPI	CMI	SR
Organic farming	1.45	1.005	0.96	96.81	0.40
Fruit Orchard Land	1.80	0.91	0.80	73.83	0.35
Pasture Land	1.28	0.36	0.37	13.48	0.43
Small scale conv. Farming	2.25	0.83	0.68	56.97	0.30
Forest land	1.32	1	1	100	0.42
Large scale Conv. Farming	1.5	0.60	0.56	34.25	0.40
Alternative Fallow& conv. Farming	1.56	0.67	0.63	42.84	0.38

*CPI* carbon pool index, *LI* lability index, *L* lability, *CMI* carbon management index, *SR* stability ratio

## Discussion

### Total soil organic matter carbon (TSOMC)

**Fig. 1:**
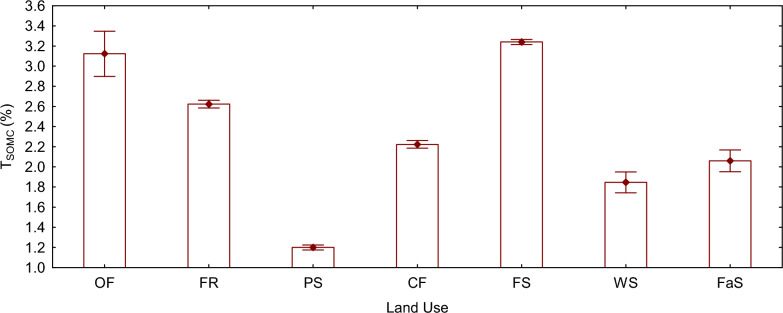
Mean plot of T_SOMC_ (%) grouped by land use type. The vertical bars denote 0.95 confidence intervals. *OF* organic farming land, *FR* fruit orchard land, *PS* pastureland, *CF* small scale conventional farming land, *FS* Forestland, *WS* large scale conventional farming land, *FaS* alternative fallow and conventional farming land, *T*_*SOMC*_ total soil organic matter carbon

Among the land use types examined, Forestland demonstrated the highest carbon pool (Fig. 1), as supported by several other studies [13, 38]. This can be attributed to the minimal disturbance of native protected forest land soils, resulting in a greater recovery of above and below-ground biomass and the formation of stable and recalcitrant carbon pools [39]. The continuous deposition of litter, coupled with a favorable environment characterized by high temperature and precipitation, promotes a high turnover rate in that region [1]. Other researchers have also reported similar findings, attributing the dominance of carbon in forest soils to factors such as continuous high carbon input materials [6], elevated soil microorganism’s activity, carbon levels around the rhizosphere [40], and soil cover that protects against erosion [41]. Moreover, organically managed lands also have exhibited distinguishable total organic matter carbon content, which can be explained by the long-term application of carbon-rich inputs, primarily in the form of stacked manure, slurry, or compost [42] and nonuse of synthetic inputs. Additionally, increased microbial activity, the adoption of best management practices (especially crop rotation length), and high biodiversity contribute to the higher total soil organic matter carbon observed in these lands [42–44]. Lastly, the higher T_SOMC_ in fruit orchards found in this study have been reported in other studies that focused on mango and lychee orchards, and were attributed to the varying quantities and qualities of organic matter input through continuous fresh litter fall, soil cover, minimum soil disturbances living organisms, and root activity (e.g., turnover and exudates) [45, 46].

In contrast, total soil organic matter carbon was lower in large-scale conventional farming lands. This can be attributed to overuse and intensive tillage practices that break down soil aggregates, exposing all soil organic matter to conducive environment for decomposition [47–49], use of synthetic inputs, as well as the failure to return crop residues to the farm [50, 51]. Surprisingly, pastureland also exhibited lower total soil organic matter carbon levels, potentially due to high grazing intensity that exposes the soil surface to harsh conditions and erosion [52], exceeding the carrying capacity of fragmented pastures [53], no management and erosion [54], which disrupt the carbon balance by creating an output that is not compensated for by sufficient input [53]. This finding contradicts the research conducted by Franzluebbers [52], which showed that grasslands exhibit higher capacity to store soil organic carbon than forest land. However, it is important to note that controlled grazing was implemented in his study area, which may have influenced the results.

### Labile fraction of soil organic matter carbon (CL)

**Fig. 2:**
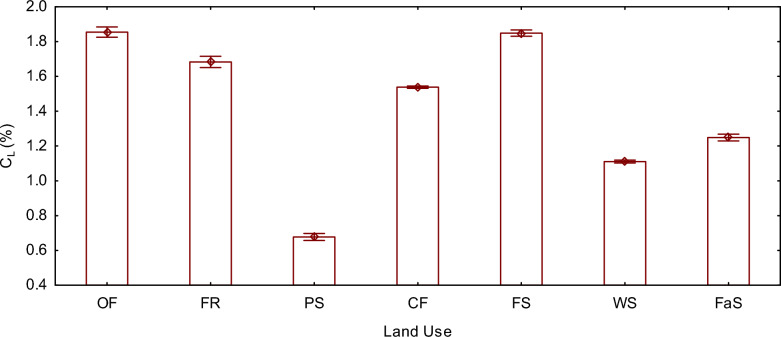
Mean plot of C_L_ (%) grouped by land use type. The vertical bars denote 0.95 confidence intervals. *OF* organic farming land, *FR* fruit orchard land, *PS* pastureland, *CF* small scale conventional farming land, *FS* Forestland, *WS* large scale conventional farming land, *FaS* alternative fallow and conventional farming land, *C*_*L*_ labile fraction of soil organic matter

**Fig. 3:**
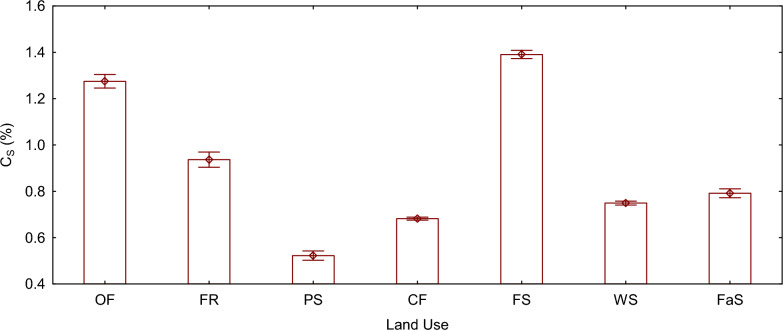
Mean plot of C_S_ (%) grouped by land use type. The vertical bars denote 0.95 confidence intervals. *OF* organic farming land, *FR* fruit orchard land, *PS* pastureland, *CF* small scale conventional farming land, *FS* Forestland, *WS* large scale conventional farming land, *FaS* alternative fallow and conventional farming land, *C*_*S*_ stable fraction of soil organic matter

**Fig. 4:**
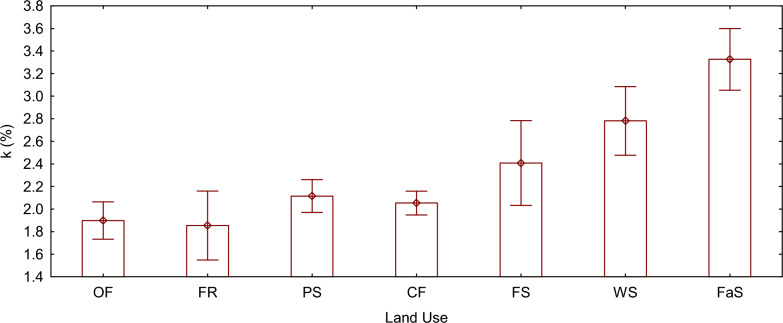
Mean plot of k (%) grouped by land use type. The vertical bars denote 0.95 confidence intervals. *OF* organic farming land, *FR* fruit orchard land, *PS* pastureland, *CF* small scale conventional farming land, *FS* Forestland, *WS* large scale conventional farming land, *FaS* alternative fallow and conventional farming land, *k* soil organic matter oxidation coefficient

Among the land use types evaluated in this study, organic farming and Forestland displayed the highest C_L_ (Fig. 2). The higher labile fraction of soil organic matter carbon in organically managed lands can be attributed to their long-term use of compost, farmyard manure, and legume cover crops, no use of synthetic inputs, which contributed to increased labile carbon levels [55, 56]. Each season, the land is tilled before applying organic fertilizers, creating favorable conditions in the soil that encourage soil microorganism activity [57]. Additionally, intercropping and crop rotation plans dominated by legumes are consistently implemented [58], while erosion protection measures, such as physical structures and grass buffers, are employed to reduce input losses [59, 60]. Similar results were reported by Francaviglia et al. [60], who demonstrated that sustainable agricultural practices, such as reduced tillage, cover cropping, and crop residue retention, are cost-effective solutions that address land degradation, food security, and climate change mitigation and adaptation by enhancing soil organic carbon sequestration and associated co-benefits. Crystal-Ornelas et al. [44] found that best management practices in organic farming led to an average increase of 18% in depth-weighted soil organic carbon concentrations and an average increase of 30% in depth-weighted microbial biomass carbon. In an experiment comparing conventional tillage to organic farming in olive groves in Mediterranean rangelands (southern Spain), Parras-Alcántara and Lozano-García [61] observed a 72% and 66% increase in total soil organic carbon in organic farming compared to conventional farming in cambisols and luvisols, respectively. Kalambukattu et al. [62] found that undisturbed land use types in the central Himalayas had higher labile soil organic matter due to the accumulation of protected carbon by soil aggregates (Figs. 3, 4)

Conversely, pastureland exhibited a lower labile fraction of soil organic matter carbon, followed by the three types of conventional farming. The lower C_L_ in pastureland may be attributed to their lack of management, reliance on self-grown (natural) grasses, and susceptibility to erosion, overgrazing, and absence of carbon-rich inputs. In their study, Becker et al. [63] found that soil organic carbon is inversely correlated with grazing frequency, and labile organic matter carbon showed a similar correlation with grazing frequency and intensity, while more stable mineral-associated organic matter carbon did not correlate with either grazing management parameter. Conant et al. [64] reported that improved grazing management, fertilization, sowing legumes and improved grass species, irrigation, and conversion from cultivation tend to lead to increased soil carbon, at rates ranging from 0.105 to more than 1 Mg C·ha^−1^·yr^−1^. Other studies also have documented relationships between soil carbon, pasture management, and grazing intensity and frequency [65–67]. Higher soil organic carbon was observed under light grazing intensity in cool-season pastures and with longer rest periods. Controlled grazing has been found to stimulate enzymes that increase microbial activities, leading to short-term mineralization and reduction in labile organic matter fraction, according to other studies [68]. Various research [39, 69, 70] demonstrate that labile organic matter, being the lighter fraction, is easily carried away in semi-arid areas with poor soil management structures. Consequently, the C_L_ was observed to be low in all land use types they evaluated and they attributed this to the fact that the labile fraction of soil organic matter is generally lower compared to mineral-associated organic carbon in the soil, as it is related to light sand size fractions that are easily transported by water erosion [71]. Furthermore, the labile soil organic matter fraction does not form organo-complexes with minerals, making it more susceptible to mineralization [72].

Moreover, the lower labile soil organic matter fraction observed in conventional farming lands may be attributed to adopted management practices such as intensive soil tillage and disturbances that break down macro and micro aggregates, disturbing the soil matrix and exposing it to higher decomposition rates and mineralization. It may also be attributed to the low use of organic fertilizers, leading to reduced carbon inputs. Several studies have found that both no-tillage and shallow tillage with residue cover had significantly higher soil organic carbon than conventional tillage without residue cover [73], while others reported that the difference between the treatments of plowing with straw return and no-tillage with straw return on total organic carbon in central China was not significant [74]. In Chitwan Valley of Nepal, no-tillage with crop residue application at the upper soil depth had distinctly higher soil organic carbon sequestration than conventional tillage with crop residue [75]. It should also be added that the effects of tillage on soil labile organic carbon vary with regional climate [1], soil condition [76, 77], residue management practice, and crop rotation [50, 59, 78, 79].

### Stable fraction of soil organic matter carbon (CS)

The stable fraction of soil organic matter was found to be higher in Forestland, which can be attributed to several factors, including the high litter input, conducive environmental conditions, rapid conversion of organic inputs and labile carbon fractions to more stable and recalcitrant forms, and the persistence of carbon under favorable conditions such as moisture and temperature, thick canopy cover, and minimal soil disturbance [1, 80]. According to Waring et al. [81], natural forests store more carbon compared to other land uses due to their complex stand structures and the accumulation of carbon belowground and in the forest floor, which also promotes the formation of more recalcitrant and stable complexes that resist decomposition. However, some studies found that grasslands had the largest soil organic carbon stock, being 1.5- and 1.8-fold higher than the stocks in forests and croplands, respectively [82], other studies demonstrated that diversity increases the temporal stability of ecosystem functions because larger species pools are more likely to contain species that can tolerate different types of perturbations [83]. Species-rich tree communities would exhibit greater temporal stability of carbon capture rates and offer higher resistance to perturbations like droughts [84] compared to monodominant plantations. Studies found that recalcitrant materials showed minimal decreases across different land use types, likely due to the inaccessibility of microorganisms and strong bonds created between soil mineral surfaces and soil organic carbon [18, 41].

In contrast, pastureland exhibited a low stable fraction of soil organic matter, which may be linked to factors such as low carbon rich inputs, high levels of overgrazing, erosion, and lack of management, leading to low herbaceous litter input, low biomass production and reduced total and stable organic carbon [6]. Land use changes can alter the equilibrium between carbon inflows and outflows in the soil, subsequently affecting soil organic carbon stocks [85]. Conversion of forest to agro-pastoral ecosystems can either increase or decrease soil organic carbon stock, depending on the type of agroecosystem implemented [86]. In conventional pastures with low levels of fertility and deterioration in soil physical properties, the low production of biomass can result in reduced total and stable carbon contents [87]. Studies by Marin-Spiotta et al. and Paul et al. have shown that the highest carbon losses among environments occur in the forest-to-pasture transition, with approximately 22.43% of carbon released from labile fractions [88, 89]. Fonte et al. [90] found that tropical pastures lacking management and experiencing degradation are linked to reduced stable soil organic matter and lower aggregation, which also affects other nutrients like phosphorus. Additionally, Rittl et al. [91] found that conversion from forest to pasture does not significantly impact the overall soil organic carbon stock, but soil organic carbon stock decreases rapidly shortly after pasture conversion to agricultural lands. Although some studies have suggested that increased photosynthesis and above-ground biomass can lead to increased carbon inputs and retention as SOM, it has also been observed that elevated primary production often results in higher biomass removal due to grazing or cutting. Various factors such as biomass removal, plant composition, compensatory growth, biomass decomposition, and carbon return in animal excreta can all influence SOM formation and decomposition. As a result, increased carbon inputs may lead to small changes, no changes, or even losses in carbon stocks. To better understand the regulation of the transfer of carbon inputs into stabilized SOM, Kirschbaum et al. [92] proposed four key points of constraint: Carbon inputs: the amount and chemical nature of carbon inputs play a crucial role in determining the potential for SOM formation and stabilization. Biomass export by grazing or cutting: the removal of biomass by grazing animals or through cutting can limit the accumulation of carbon in the soil. Retention into different pools for SOM formation: the effects of changes in carbon inputs can vary depending on how carbon is retained in different pools, influencing SOM formation. Carbon loss from SOM decomposition and other factors: the rate of carbon loss through SOM decomposition and other processes also affects the overall carbon stocks in the soil. These factors interact in complex ways, leading to a dynamic balance between carbon inputs, retention, and loss in the soil, ultimately influencing the stability and long-term sequestration of soil organic carbon.

### Oxidation speed constant (k)

To understand the quality and decomposition rates of labile soil organic matter fractions in different land use types, it was essential to study the kinetics of soil carbon oxidation [8, 22]. In this study, the alternative fallow and conventional farming land exhibited higher oxidation compared to other land use types. This can be attributed to factors such as a highly-exposed soil, rotation for crops like legumes (such as soybean, common bean, cluster bean, mungbean, and blackgram) and oilseeds (such as Rapeseed, Sesame, and sunflower) alternating with fallow periods. Conventional farming practices, specific crop choices, and farming systems contribute to favorable soil structural conditions, which, when combined with environmental factors (moisture and temperature) in the study region, increase the decomposition rate [93–95]. Legume-based cropping systems, particularly those involving legumes rotations, have been found to significantly impact soil organic matter carbon levels over both short and long durations. Legumes, characterized by their low carbon-to-nitrogen ratio, contribute easily degradable residues that enhance soil organic matter pools [96]. Additionally, they stimulate soil biological activity, improve soil structure, enhance soil aeration, and increase water-holding capacity [97]. Alternative leguminous fallows with other crops production have also been shown to improve soil quality, as indicated by the status of labile organic matter [98].

Fruit orchard lands demonstrated low oxidation rate compared to other soil use types. This can be attributed to various factors, such as the tougher structure, higher lignin, different polyphenols content, and presence of other chemical compounds with antimicrobial properties in fruit tree leaves and other parts, including those of lychee and mango trees [46].. However, their slower breakdown contributes to soil organic matter accumulation and nutrient cycling, making them valuable components in ecosystem processes [99, 100]. Musvoto et al. [101] assessed changes in lignin, cellulose, hemicellulose, polyphenols, and nutrient release in mango and miombo woodland, Miombo litter had higher initial concentrations of N, P, S, Mg, and lignin, and lower polyphenol contents than mango litter. Mass loss was faster in miombo than in mango litter and the rate of lignin loss was higher in miombo than in mango litter. Total polyphenols could not be detected in either litter type after two months. Another study by Tarrsini and Ng [102] found that mango fruit derivatives are rich in lignin, cellulose, hemicellulose, and polyphenols, making them challenging to decompose. The natural recalcitrance of lignocellulosic plant cell walls gives resistance to enzymatic hydrolysis, and proposed a pretreatment method to disrupt the complex lignin barrier and increase accessibility to lignocellulosic biomass holocellulose (cellulose and hemicellulose) prior to enzymatic hydrolysis to ensure effective bioethanol production. Regarding rates of decomposition and nutrient mineralization of leaf litter from different orchards under hot and dry sub-humid climates, Naik et al. [103] found that leaf litter of mango and guava decomposed more rapidly than that of lytchee (litchi), with decay constants of 3.22, 1.33, and 0.62 yr^−1^, respectively. Polyphenols were lost more rapidly followed by cellulose, lignin, and ligno-cellulose throughout the decomposition period. Nitrogen was released faster in mango and guava, while potassium was released faster in mango followed by guava and then lytchee (litchi).

### Stability ratio (SR)

The stability of the stable fraction of Soil Organic Matter was evaluated using a parameter called the stability ratio (SR). This ratio indicates the percentage of the stabilized fraction compared to the total organic matter carbon present in the sample. It serves as a measure of how well the organic matter in the soil is protected from decomposition. A higher SR value suggests a stronger degree of stabilization, indicating that a larger portion of organic carbon is less susceptible to rapid breakdown which has the potential to contribute to long-term carbon storage in the soil [22–24].

Surprisingly, despite having a lower total soil organic matter carbon content, pastureland has exhibited a higher stability ratio compared to other land use types. This unexpected finding suggests that even though pastureland may contain less organic matter overall, the organic matter present in these areas is more resistant to decomposition and remains more stable in the soil. The reasons for this higher stability ratio in pastureland could be attributed to various factors, such as the reduced soil disturbance and the presence of natural grasses in pastures that may contribute to a balanced decomposition process and the preservation of stable organic matter fractions in the soil. This may also be attributed to the fact that pastureland do not receive any management. The grasses are natural, with no carbon inputs, no tillage, or any other kind of soil disturbances apart from overgrazing. In their study, Barral et al. [104] found that pasture soils have more favorable structural properties than cultivated soils, showing lower bulk density, higher porosity, and water retention. The pasture soil also exhibits a higher mean aggregate diameter and aggregate stability against mechanical agitation in water, as well as lower soil loss under simulated rainfall. This increased structural stability of pasture soil could be attributed to its higher stable fraction of soil organic matter content [105]. In addition to the great biodiversity and root exudates in pastures, the reduced soil disturbance allows the stable SOM to be less exposed to conditions that could accelerate its decomposition, allowing it to accumulate over time [106]. The presence of grazing animals in pastures also influences the cycling of organic matter and nutrients, promoting a more balanced decomposition process and further contributing to SOM carbon stabilization [107]. Overall, the combination of continuous root turnover, high-quality litter, reduced disturbance, grazing activity, and greater biodiversity in pastures collectively contributes to the higher SOM carbon stabilization observed in these land use type compared to forests, fruit orchards, and agricultural lands [52, 66].

In contrast, conventional farming generally exhibited lower stable organic matter fractions compared to other land use types due to intensive tillage practices that disrupt soil structure and accelerate organic matter exposure and decomposition [108]. Additionally, reliance on synthetic fertilizers and limited incorporation of organic residues further hinder stable organic carbon buildup [109]. Monoculture and reduced biodiversity in conventional farming also limit diverse plant inputs and soil microbial communities responsible for organic matter stabilization [1]. Erosion caused by farming practices can remove topsoil and its stable organic matter fraction, exacerbating the decline in soil carbon [110]. In contrast, land use types such as grasslands, pastures, and forests benefit from natural processes and diverse plant inputs, contributing to higher stable organic matter fractions [63]. Adopting sustainable practices in agriculture, such as conservation agriculture and reduced tillage, can help improve stable organic matter fractions in agricultural soils and enhance carbon sequestration for long-term sustainability [59, 61].

### Carbon management index (CMI)

As evident from the current study, different land use types have led to varying levels of soil quality degradation. However, the extent of damage and the necessary management actions to improve degraded land remain unanswered. To address these issues, the carbon management index has been calculated to provide insights into carbon depletion (Table 1). CMI offers an integrated measure of both the quantity and quality of soil organic carbon. Unlike a single measure such as total soil organic carbon concentration, CMI serves as a more sensitive indicator of the rate of change of SOC in response to soil management practices, and has been suggested as a useful technique for assessing soil fertility [5, 22, 111].

To calculate the extent of damage in different land use types, the native forest was used as a reference ecosystem due to its higher total soil organic matter carbon pool, and from the fact that it is conserved, less disturbed, and before the adoption of other land use types the whole area was covered by bush and forested. CMI is based on this native ecosystem and has no definite standards [6]. Higher CMI values signify better soil carbon buildup, while lower values indicate carbon degradation [5]. Comparatively, high CMI values were observed in Forestland, organic farming land, followed by fruit orchard land, which can be attributed to their long-term sustainable management practices with higher carbon-rich inputs, less oxidative environment, thicker canopy and litter, and low erodibility [5]. Consequently, forest, organic farming, and fruit lands perform better in sustaining soil carbon compared to other land use types. On the other hand, lower CMI values in all types of conventional farming and pastureland indicate reduced potential for soil carbon sequestration compared to forests, organic farms, and fruit orchards at study area. These findings are in agreement with Sainepo et al. [6] and also highlight the need for implementing carbon improvement strategies to address the significant carbon depletion in grasslands and bare lands when compared to forest or shrub lands. Moreover, Vieira et al. [112] studied the CMI in agricultural land use and proposed that introducing winter vetch and, especially, summer legume cover crops (cowpea and pigeon pea), or applying fertilizer-N, could improve the capacity of the management system and promote soil quality in subtropical acrisols. Additionally, Jagadesh et al. [13] findings highlight the pronounced carbon depletion in agricultural lands and tea ecosystems compared to forest ecosystems. This emphasizes the urgent and imperative need for immediate implementation of carbon management strategies in agricultural and tea lands. By adopting such strategies, we can enhance the carbon sequestration potential of these lands, work towards achieving land degradation neutrality, and ultimately improve the overall carbon footprints of these ecosystems. Taking prompt action in this regard is crucial for mitigating the negative impacts of carbon depletion and fostering a more sustainable and resilient environment.

## Conclusion

This study conducted in the Bharatpur Catchment, Chitwan District, Nepal, highlights the substantial impact of land use on soil organic carbon pools. It underscores the critical importance of comprehending soil organic matter dynamics amid changing land use patterns and advocates for the use of the Carbon Management Index (CMI) and Stability Ratio (SR) as pivotal indicators in sustainable land management, fostering soil health, environmental sustainability, and resilience. Organic farming, forests, and fruit orchards exhibit elevated labile organic matter fractions (C_L_) and total soil organic carbon (T_SOMC_), reflecting sustainable practices, while conventional agricultural lands register lower C_L_ and T_SOMC_ levels attributed to less favorable practices and input types. The stable fraction (C_S_) consistently lags behind C_L_, suggesting historical deforestation and overuse. The CMI analysis underscores severe degradation in conventional farming and pastures lands, necessitating immediate action to curb above-ground biomass harvesting, overgrazing, and promote soil conservation practices for bolstering soil organic carbon accumulation. Soil management strategies within agricultural lands should prioritize augmenting both labile and stable organic matter fractions to enhance long-term soil fertility.

This study underscores the imperative need to fathom soil organic matter dynamics in the context of evolving land use. CMI and SR serve as invaluable tools for gauging soil degradation or improvement, offering guidance for sustainable land management. Armed with this knowledge, proactive measures can be taken to conserve and enhance soil health, thereby advancing overall environmental sustainability and resilience within the Bharatpur Catchment, Chitwan District, Nepal. Ongoing research and analysis will be instrumental in continuously monitoring evolving trends and comprehending the underlying mechanisms behind these intriguing observations, with far-reaching implications for soil health and carbon cycling across diverse land use categories.

In practical terms, if resources are limited for detailed soil assessments, prioritizing the Carbon Management Index (CMI) can provide a comprehensive view of soil quality and its response to land use. Nepal especially in the study region should also consider sustainable land management practices like organic farming and afforestation to improve soil health and carbon storage. When planning land use, it's essential to strike a balance between agriculture, forestry, and other uses to preserve soil health and biodiversity while meeting agricultural needs. While there is no need to outright ban conventional farming, it is crucial to implement soil conservation methods and reduce soil disturbances associated with conventional practices. In essence, the choice between CMI and SR testing depends on goals and resources, with a focus on sustainable land management and balanced land use distribution to ensure soil health and overall environmental sustainability.

## Data Availability

The manuscript contains all the necessary data, and we are prepared to furnish any additional information upon request.
